# Deletion of *GPR30* Drives the Activation of Mitochondrial Uncoupling Respiration to Induce Adipose Thermogenesis in Female Mice

**DOI:** 10.3389/fendo.2022.877152

**Published:** 2022-05-03

**Authors:** Jing Luo, Yao Wang, Elizabeth Gilbert, Dongmin Liu

**Affiliations:** ^1^ Guangdong Provincial Key Laboratory of Food, Nutrition and Health, Department of Nutrition, School of Public Health, Sun Yat-sen University, Guangzhou, China; ^2^ Department of Human Nutrition, Foods and Exercise, College of Agricultural and Life Sciences, Virginia Tech, Blacksburg, VA, United States; ^3^ Department of Animal and Poultry Sciences, College of Agricultural and Life Sciences, Virginia Tech, Blacksburg, VA, United States

**Keywords:** mitochondrial respiration, thermogenesis, GPR30, female mice, fatty acid oxidation

## Abstract

Thermogenic adipocytes possess a promising approach to combat obesity with its capability promoting energy metabolism. We previously discovered that deletion of *GPR30* (GPRKO), a presumably membrane-associated estrogen receptor, protected female mice from developing obesity, glucose intolerance, and insulin resistance when challenged with a high-fat diet (HFD). *In vivo*, the metabolic phenotype of wild type (WT) and GPRKO female mice were measured weekly. Acute cold tolerance test was performed. *Ex vivo*, mitochondrial respiration of brown adipose tissue (BAT) was analyzed from diet-induced obese female mice of both genotypes. *In vitro*, stromal vascular fractions (SVF) were isolated for beige adipocyte differentiation to investigate the role of GPR30 in thermogenic adipocyte. Deletion of *GPR30* protects female mice from hypothermia and the mitochondria in BAT are highly energetic in GPRKO animals while the WT mitochondria remain in a relatively quiescent stage. Consistently, *GPR30* deficiency enhances beige adipocyte differentiation in white adipose tissue (WAT) and activates the thermogenic browning of subcutaneous WAT due to up-regulation of UCP-1, which thereby protects female mice from HFD-induced obesity. GPR30 is a negative regulator of thermogenesis, which at least partially contributes to the reduced adiposity in the GPRKO female mice. Our findings provide insight into the mechanism by which GPR30 regulates fat metabolism and adiposity in female mice exposed to excess calories, which may be instrumental in the development of new therapeutic strategies for obesity.

## Introduction

The escalation in the prevalence of obesity worldwide has nearly tripled since 1975 and continues to rise rapidly (1). Obesity increases the risks of a series of severe health conditions, including type 2 diabetes, cardiovascular disease, musculoskeletal disease, Alzheimer’s disease, depression, and some types of cancer (2–4). Obesity might not be a fatal condition; however, it impairs life quality of individuals and becomes a major public health burden because of its complex pathophysiology. Obesity can be caused by excessive energy intake, reduced energy expenditure, or a combination of both (5, 6). Individuals with obese or overweight conditions often have difficulties restoring energy homeostatic capabilities, even after short-term behavioral changes or medical interventions to lose weight (4). Although mounting efforts to promote healthy eating habits and more physical activity to enhance energy expenditure can be helpful at the societal level, these attempts are not sufficient for people already living with a high body weight, particularly when there is limited compliance with prescribed behavioral changes. Therefore, impacting energy homeostasis without altering energy intake or physical activity would pose a challenging yet promising intervention option to combat obesity.

The white adipose tissue (WAT) is the major energy storage tissue (6, 7), whereas brown adipose tissue (BAT) dissipates excess energy to generate heat through the action of mitochondrial uncoupling protein-1 (UCP-1), thereby protecting against hypothermia and obesity (8). Results from positron emission tomography (PET) scanning showed that human adults possess depots of BAT, which share physiological similarity with rodent beige fat rather than the classical rodent interscapular BAT (9, 10). Though recruited from subcutaneous WAT, beige adipocytes contain a relatively high density of mitochondria and UCP-1 (11), which are highly inducible in response to β adrenergic receptors stimuli, including cold and overfeeding (10, 12).

The stimulated catabolism of lipids and carbohydrates in thermogenic adipocytes enhances metabolic homeostasis (13, 14) and this thermo-promoting response is closely influenced by G protein-coupled receptors (GPCRs) (15–17). GPCRs are the largest family of cell membrane-associated receptors in the human genome (18). GPCRs have a signature structural similarity containing seven transmembrane domains, one extracellular amino terminus, and one intracellular carboxyl terminus that transduce cellular signaling (19). Metabolic substrates oxidation fuels the mitochondrial futile cycles (20) and the mitochondrial UCP-1-mediated respiration dissipates energy chemicals to ultimately generate heat in thermogenic adipocytes (21). GPCRs are involved in the stimulation of thermogenic beige adipocyte recruitment in the subcutaneous WAT (10, 22). We previously discovered that deletion of G protein-coupled receptor 30 (*GPR30*), a recognized membrane-associated estrogen receptor (23), protected female mice from high-fat diet (HFD)-induced obesity (24). While the knockout of *GPR30* (GPRKO) didn’t alter the amount of food intake, GPRKO female mice had significantly higher body temperatures as compared with HFD-fed WT female mice, suggesting that there is a difference in thermogenesis that likely contributes to the reduced fat accumulation over time in GPRKO female mice fed the HFD. Thus, we tested in the present study whether deletion of *GPR30* may directly modulate thermogenic capacity in adipose tissue, thereby exerting protective effects against diet-induced obesity.

## Materials and Methods

### Materials

Dexamethasone, 3-isobutyl-1-methylxanthine (IBMX), insulin, triiodothyronine (T3), indomethacin, rosiglitazone, forskolin were purchased from Sigma-Aldrich (St. Louis, MO, USA). DMEM-high glucose, bovine serum albumin (BSA), and fetal bovine serum (FBS) were from HyClone (Logan, UT, USA). Antibiotic Antimycotic Solution (Ab/Am) was from Corning (Manassas, VA, USA) and Collagenase was from Worthington (Lakewood, NJ, USA). Antibody for UCP-1 (AB3036) was from Millipore (Burlington, MA, USA). Antibody for Tubulin was from Santa Cruz Biotechnology (Santa Cruz, CA, USA). Nitrocellulose membranes and protein assay kits were from Bio-Rad (Hercules, CA, USA). Cyclic AMP ELISA kit was from Cayman Chemical Co (Ann Arbor, MI, USA). All other chemicals and enzymatic kits are indicated below.

### Animals

All protocols for the following animal experiments were approved by the Institutional Animal Care and Use Committee at Virginia Tech. GPR30 heterozygous mice on a 129 background were kindly provided by Dr. Deborah J. Clegg (UT Southwestern Medical Center, TX). Homozygous GPRKO and their WT littermates were generated by mating heterozygous mice, and genotyped using PCR. All mice were housed under constant temperature (22-24°C) with a 12h light/dark cycle and free access to standard chow diet (STD, D06072701, Research Diets, New Brunswick, NJ, USA) and water.

### Mitochondrial Respiration Measurements

Both WT and GPRKO female mice (n=3) at 12 weeks of age were housed separately and fed a 58% HFD (Research Diets Inc., New Brunswick, NJ, USA) *ad libitum* for one month to induce adiposity. Then the measurements of mitochondrial respiration of the whole adipose tissue was assessed as instructed (25, 26) with modifications. Briefly, approximately 10 mg of BATs was isolated from the same interscapular area of each animal and the fat pads were cut into small pieces and placed into the Agilent Seahorse (Santa Clara, CA) XF24 tissue culture plate. After washing with the assay buffer (Seahorse XF base medium) three times, mitochondrial respiration *via* oxygen consumption rate (OCR) and extracellular acidification rate (ECAR) were measured in the XF24 plate reader following the established protocol (25) to determine the mitochondrial response: 8 µg/mL oligomycin, 8 µM FCCP (mitochondrial oxidative phosphorylation uncoupler), 12 µM antimycin A (AA), and 3 µM rotenone (Rote). Basal OCR was determined by five consecutive measurements after tissue equilibration and the overall OCR was calculated by averaging the total 20 measurements. Data were normalized to the wet weight of fat pads.

### Fatty Acid Oxidation Measurements

Fatty acid oxidation in the interscapular BAT from WT and GPRKO female mice were assessed by measuring and summing ^14^CO_2_ production and ^14^C-labeled acid-soluble metabolites from the oxidation of [1-^14^C] palmitic acid from Perkin Elmer (Waltham, MA) as previously described (24). Both WT and GPRKO female mice used for this study were fed a HFD for one month starting 12 weeks of age.

### Adipose Tissue Stromal Vascular Fraction (SVF) Cells Isolation

SVF from WAT of WT and GPRKO female mice were isolated as instructed (27). Briefly, subcutaneous white fat pads were washed in Krebs-Ringer bicarbonate buffer (KRB) supplemented with 1% BSA then quickly transferred to the collagenase solution for approximately 50 min at 37°C with 10 seconds shaking at 10 minutes interval apart. After the collagenase digestion, stromal cells were separated by centrifugation twice at 300 rcf for 5 min and washed twice with prewarmed PBS supplemented with 1% BSA afterwards. SVF were then seeded for subsequent experiments.

### Intracellular Cyclic AMP (cAMP) Measurements

SVF cells isolated from obese WT and GPRKO female mice were seeded in 6-well plates at 1 x 10^6^ cells/well. On the next day after seeding, culture medium was changed to KRB for 20 min and the SVF cells were then treated with vehicle or forskolin (5 µM) for 15 min. The intracellular cAMP contents were measured by using a cyclic AMP ELISA kit as previously described (28). Data were normalized to the cellular protein concentration in the same samples.

### CRE-Luciferase Activity Measurements

To determine whether GPR30 affects cAMP-regulated transcription, CRE-luciferase (CRE-luc) activity in SVF cells were measured as previously described (29). Briefly, subcutaneous SVF cells from WT and GPRKO female mice were seeded in a 12-well plate until approximately 60% confluence. Then cells were co-transfected with 0.25 μg CRE-luc reporter plasmid and 5 ng pRL reporter control vector per well using Lipofectamine 2000 transfection reagent (Invitrogen, CA, USA). After overnight transfection as instructed, stromal cells from HFD-fed WT and GPRKO female mice were starved with serum-free medium for 4 hours. After starvation, stromal cells were changed to complete culture medium (DMEM supplemented with 10% FBS and 1% Ab/Am) with or without forskolin (5 μM) for 16 hours. Luciferase activity was determined using a dual-luciferase assay system (Promega, WI, USA) and normalized to pRL renilla activity.

### Beige Adipocyte Differentiation

Beige adipocyte differentiation was induced by treating confluent but not packed stromal vascular cells isolated from subcutaneous WAT with DMEM medium containing 10% FBS, 1% Ab/Am, 125 µM indomethacin, 5 µM dexamethasone, 0.5 mM IBMX, and 0.5 µM rosiglitazone. Two days after induction, cells were maintained in medium containing 10% FBS, 1% Ab/Am, 5 µg/ml insulin, and 1 nM T3 for an additional two days, and then cultured in complete medium for 3-4 days until the multilocular droplets formed and filled with lipids (30). The triglycerides stored in the droplets of beige adipocytes were quantified by an Oil Red O staining assay as previously described (24). To investigate the role of GPR30 in SVF differentiation by using synthetic compounds, the GPR30 specific agonist G-1 (100 nM) and antagonist G15 (5 μM) were added together with the induction cocktail to both WT and GPRKO cells during beige adipocyte differentiation. Total RNAs were collected after differentiation and thermogenic genes were analyzed.

### Cold-Induced Thermogenesis *In Vitro*

Stromal vascular cells were isolated from subcutaneous WAT of WT and GPRKO female mice and then treated with beige adipocytes differentiation cocktail as described above. Total RNAs were collected on the initiation day and termination day of differentiation with or without cold exposure (31°C, 5% CO_2_) for four hours to investigate the effect of GPR30 in cold-stimulated thermogenesis (31, 32).

### Cold Tolerance Test

Starting at 10 weeks of age, the body weight (BW), food intake, and rectal body temperature of both WT and GPRKO female mice were measured weekly (n=7-10). The acute cold tolerance test was performed as described (31) at week 8. Briefly, the rectal body temperature was measured before moving mice from a normal room temperature (RT) into a 4°C room (Cold) with *ad libitum* access to STD diet and water for 4 hours. The condition and behavior of mice were constantly observed during the test. The rectal temperature of mice was measured at the end of the test, and then mice were transferred to the RT environment.

### Western Blot

Subcutaneous WAT and interscapular BAT lysates with equal amounts of protein from WT and GPRKO female mice were subjected to immunoblot analysis as previously described (29, 33). The immune-reactive proteins were detected by chemiluminescence and quantified using a ChemiDoc™ Touch Imaging System (Bio-Rad, Hercules, CA, USA). The relative protein levels were normalized to those of the housekeeping protein and compared to the WT group.

### Real-Time Quantitative PCR

Total RNA was extracted from adipose tissues and beige adipocytes using TRI reagent (Molecular Research Center, OH) and reverse-transcribed using GoScript™ Reverse transcriptase and random primers (Promega, WI). Amplification reactions were performed on an Applied Biosystems^®^ 7500 Fast Real-Time PCR System as we previously described (34). Data were analyzed by the RQ=2^−ΔΔCt^ method. The primers used were: *Ucp-1* (5’-AGCCGGCTTAATGACTGGAG-3’ and 5’-TCTGTAGGCTGCCCAATGAAC-3’), *Prdm16* (5’-CAGCACGGTGAAGCCATTC-3’ and 5’-GCGTGCATCCGCTTGTG-3’), *mCidea* (5’-TGCTCTTCTGTATCGCCCAGT-3’ and 5’-GCCGTGTTAAGGAATCTGCTG-3’), and *18S RNA* (5’-ACCTGGTTGATCCTGCCAGTAG-3’ and 5’-TTAAiTGAGCCATTCGCAGTTTC-3’).

### Statistical Analyses

Data were analyzed using the software JMP (Version 12, SAS Institute, Cary, NC, USA). Group differences were compared using one-way analysis of variance (ANOVA) followed by Tukey’s test, with p<0.05 considered significantly different. Data are presented as mean ± SE.

## Results

### Deletion of GPR30 Promotes Cellular Respiration in BAT

**Figure 1:**
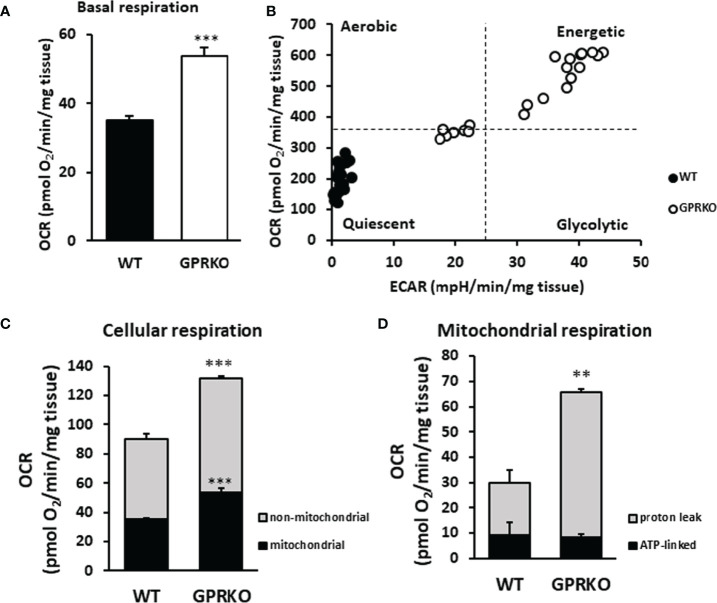
Deletion of GPR30 promotes cellular respiration in BAT. BAT from three WT and GPRKO female mice, respectively, were analyzed using the XF24 Islet Capture Microplate as described in the Materials and Methods. **(A)** Basal OCR were calculated after the equilibration of tissues and before oligomycin injection. **(B)** The overall OCR and ECAR were plotted to represent the mitochondrial metabolic states. **(C)** Cellular and **(D)** mitochondrial respiration was calculated. Data are normalized to wet weight of fat pads and represented as means ± SE (n=3). **p<0.01. ***p<0.001.

Our previous work, using both *in vivo* and *in vitro* approaches, demonstrated that deletion of GPR30 protected female mice from HFD-induced obesity and the GPRKO animals had higher body temperature (24). To further explore how GPR30 deficiency exerts a metabolic promoting effect, we measured mitochondrial oxygen consumption rate (OCR) and extracellular acidification rate (ECAR) in BAT from the same area of each animal using the Agilent Seahorse metabolic analysis. As shown in **Figure 1A**, basal respiration in BAT was highly promoted in GPRKO females as compared to that in WT littermates. Interestingly, FCCP-stimulated maximal respiration were the same between WT and GPRKO mice (data not shown). To address the difference in mitochondrial metabolic phenotype of WT and GPRKO mice, the bioenergetics mapping was plotted using the overall OCR and ECAR (35, 36). As seen in **Figure 1B**, all measurements of WT animals fell into the quiescent quadrant, whereas the majority of the GPRKO measurements fell under the energetic quadrant with higher OCR and ECAR, suggesting that deletion of GPR30 promotes the mitochondrial energetic phenotype.

To determine mitochondrial and non-mitochondrial respiration (37), rotenone (complex I inhibitor) and antimycin A (complex III inhibitor) were added at the final stage of the cellular respiration analysis experiment as described (38). As shown in **Figure 1C**, GPRKO female mice had significantly higher mitochondrial and non-mitochondrial respiration, respectively. To determine the amount of oxygen consumption driven by mitochondrial inducible proton leak, the ATP synthase inhibitor oligomycin was added. As shown in **Figure 1D**, the ATP-linked mitochondrial respiration was similar between WT and GPRKO female BAT; however, the inducible mitochondrial proton leak significantly increased in the HFD-fed GPRKO female BAT as compared with WT littermates, suggesting a more efficient ATP utilization and a lower efficiency of mitochondrial coupling respiration while elevated uncoupled respiration possibly mediated by UCP-1 in the absence of GPR30.

### Deletion of GPR30 Affects Fatty Acid Oxidation in BAT and Boosts cAMP Release in WAT-Derived SVF

**Figure 2:**
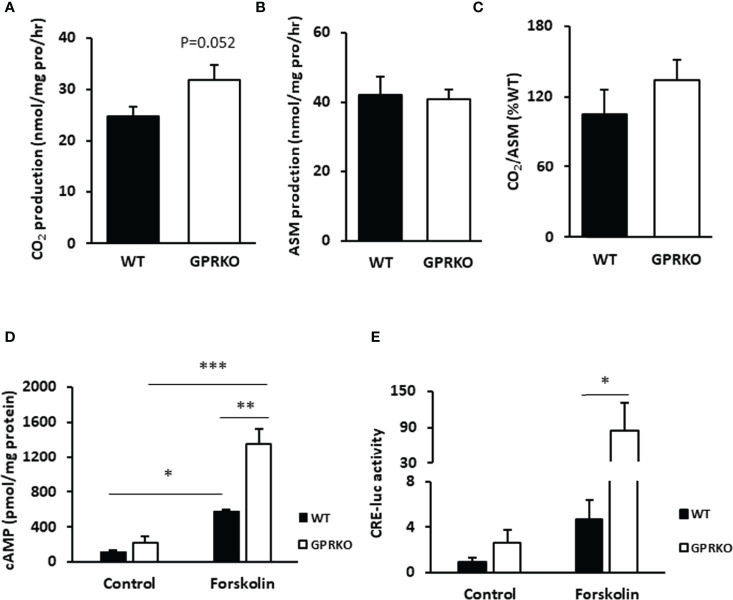
Deletion of GPR30 affects fatty acid oxidation in BAT and boosts cAMP release in WAT-derived SVF. **(A)** CO_2_ production and **(B)** acid soluble metabolites (ASM) production of BAT were measured as described in Materials and Methods. **(C)** Substrate oxidation efficiency were calculated as the ratio of CO_2_ to ASM production. **(D)** Primary SVF from subcutaneous WAT of WT and GPRKO were isolated from HFD-fed mice. The intracellular cAMP production was measured as described in Materials and Methods. Adenylyl cyclase agonist forskolin alleviated cAMP production level to 2.34 fold higher in HFD-fed GPRKO cells than that of WT. **(E)** CRE-luciferase activity was alleviated in GPRKO cells when treated with adenylyl cyclase agonist forskolin. Data are represented as means ± SE (n=3-4). *p<0.05; **p<0.01; ***p<0.001.

Since the increased proton leak respiration in GPRKO female BAT may have resulted from an increased substrate oxidation (39), we isolated BAT from WT and GPRKO female mice that were fed the HFD for 4-weeks and assessed substrate oxidation. As shown in **Figure 2A**, GPRKO female mice tended to have higher CO_2_ production in BAT as compared with the WT littermates (31.76 ± 3.01 mmol/mg pro/hr vs. 24.76 ± 1.86 mmol/mg pro/hr, respectively, p=0.052), whereas the production of acid soluble metabolites (ASM) in BAT was similar between WT and GPRKO female mice (**Figure 2B**). Hence, the efficiency of substrate oxidation in BAT were not significantly altered by the deletion of GPR30 (**Figure 2C**).

As reported before, the well-known second messenger cAMP activates protein kinase A (PKA), which activates a lipase that hydrolyzes triglycerides into glycerol and free fatty acids, the latter being a direct substrate for mitochondrial thermogenesis (40, 41). As shown in **Figure 2D**, the stromal cells from HFD-fed GPRKO female mice tended to produce more cellular cAMP as compared with the cells from HFD-fed WT littermates. The cAMP content increased 2.34-fold in GPRKO cells over the WT cells, when treated with the adenylyl cyclase agonist forskolin (p=0.006). Consistently, the cAMP-mediated transcriptional activity in GPRKO stromal cells as determined by a cAMP-responsive element (CRE) driven luciferase activity assay was greater than in WT cells (**Figure 2E**).

### Deletion of GPR30 Promotes Mitochondrial Uncoupling Respiration in BAT

**Figure 3:**
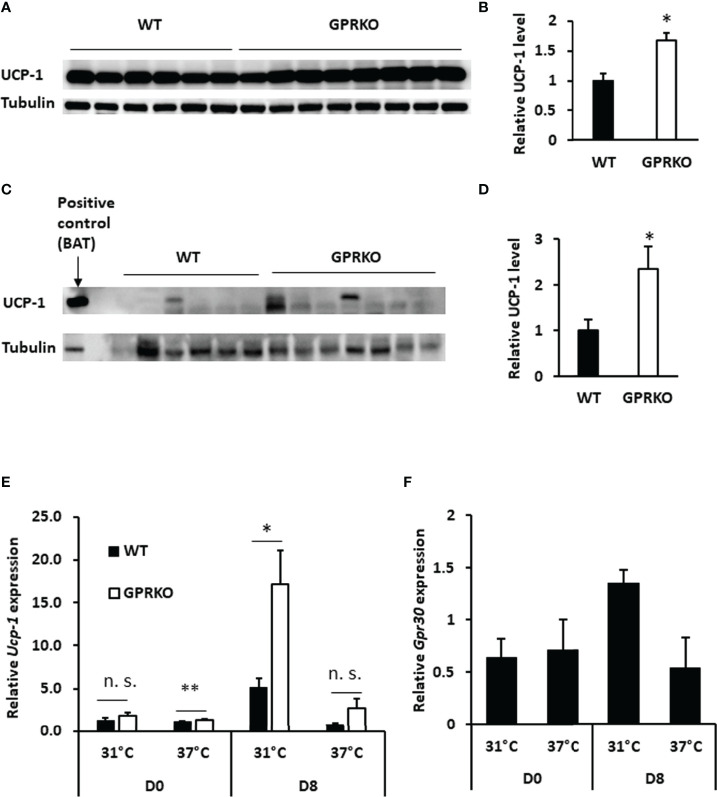
Deletion of GPR30 upregulates UCP-1 in response to overfeeding or cold exposure. **(A)** Western blot analysis of UCP-1 and tubulin loading control from BAT and **(B)** densitometric analysis of UCP-1 protein levels normalized to tubulin. **(C)** Western blot analysis of UCP-1 and tubulin loading control from subcutaneous WAT and **(D)** densitometric analysis of UCP-1 protein levels normalized to tubulin. All data are presented as means ± SE (n=6-8). *p<0.05. Total RNAs were collected after cold challenge as described in Materials and Methods and the relative gene expression of **(E)** *Ucp-1* and **(F)** *Gpr30* were analyzed by RT-qPCR. Data are normalized to WT and represented as means ± SE (n=3). *p<0.05. **p<0.01. n.s., not significant.

As discussed above, GPRKO BAT had lower efficiency of coupling respiration but the overall mitochondrial OCR was higher than that of WT, indicating that deletion of GPR30 may promote inducible mitochondrial uncoupling respiration. Accordingly, interscapular BAT was isolated from WT and GPRKO obese female mice to assess the effect of GPR30 on thermogenesis. As shown in **Figures 3A, B**, the relative UCP-1 protein levels in BAT were significantly higher in GPRKO animals as compared to WT female mice. The stimulated UCP-1 activity within subcutaneous WAT is shown to improve metabolic phenotypes (42–44), thus protein lysates from subcutaneous WAT of WT and GPRKO female mice were probed against UCP-1 and it was only detectable in one of the WT samples while half of the GPRKO samples displayed relatively high levels of UCP-1 (**Figures 3C, D**).

Deletion of GPR30 increased *Ucp-1* gene expression in the isolated SVF before treating cells with differentiation cocktail (Day 0, D0). The gene expression of *Ucp-1* in GPRKO group tended to be higher as compared to WT beige adipocytes on the last day of differentiation (Day 8, D8). Interestingly, *Ucp-1* abundance in GPRKO cells elevated approximately three times higher than that of WT after 4 hours of cold exposure (**Figure 3E**). Interestingly, the expression of *Gpr30* displayed a similar pattern in WT SVF-differentiated adipocytes after cold exposure (**Figure 3F**), suggesting a possible correlation between GPR30 and the mitochondrial uncoupling proteins in thermogenic adipocytes.

### Deletion of GPR30 Promotes Beige Adipocyte Differentiation and Activates the Thermogenic Browning of Subcutaneous WAT

**Figure 4:**
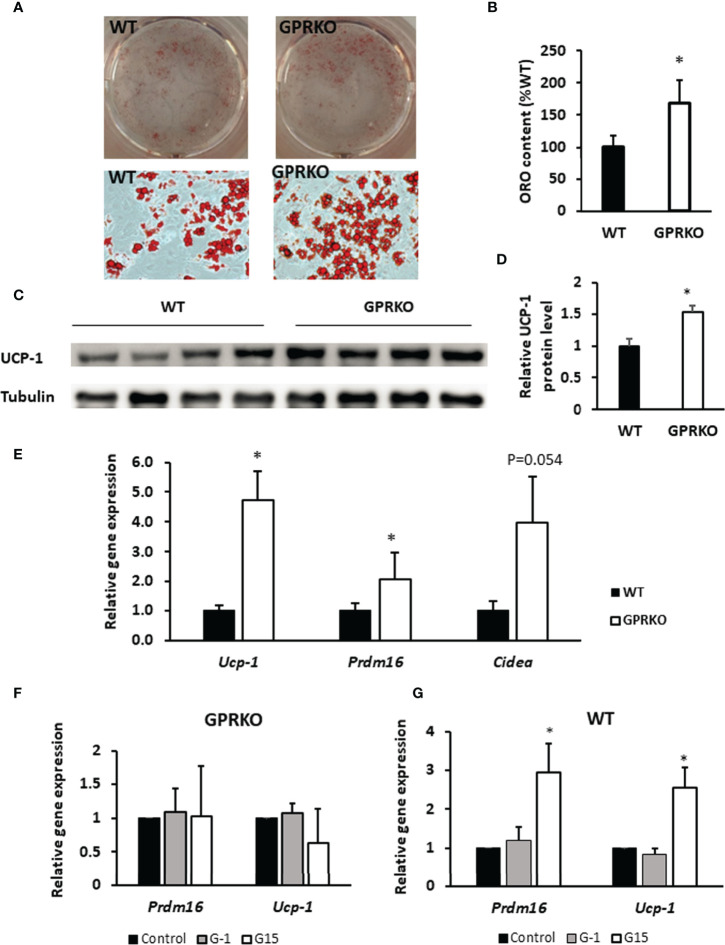
Deletion of GPR30 promotes beigeing of WAT. **(A)** The triglycerides stored in the multilocular lipid droplets were visualized using Oil Red O staining as indicated respectively. **(B)** The Oil Red O stain content were extracted and normalized to WT. Data are represented as means ± SE (n=3). **(C)** Western blotting against UCP-1 in mature beige adipocytes. Tubulin served as loading control. Data are normalized to **(D)** WT and represented as means ± SE (n=4). **(E)** mRNA levels for brown fat-selective genes *Ucp-1*, *Cidea*, and *Prdm16* were analyzed by RT-qPCR. Data are normalized to WT and represented as means ± SE (n=7). The relative gene expression of *Ucp-1* and *Prdm16* in **(F)** GPRKO- and **(G)** WT-derived cells treated with G-1 and G15 were analyzed by RT-qPCR. Data are normalized to control and represented as means ± SE (n=3). *p<0.05.

To characterize the potential role of GPR30 in browning of WAT, we isolated the primary SVF from subcutaneous WAT and treated with beige adipocyte induction cocktail containing rosiglitazone, indomethacin, dexamethasone, and IBMX (30). As seen in **Figures 4A, B**, stromal cells isolated from GPRKO subcutaneous fat displayed an increased beige differentiation efficiency, about 68% higher, than that of the WT control. Consistently, the protein expression of UCP-1 in GPRKO-isolated SVF-differentiated beige adipocytes was 53% higher than that of the WT group (**Figures 4C, D**). The expression of the brown/beige fat selective genes (*Ucp-1*, *Prdm16*, and *Cidea*) (45–48) were 2-4 fold higher in the differentiated GPRKO beige adipocytes than that of WT (**Figure 4E**). In addition, we treated the primary stromal cells with GPR30 specific agonist G-1 and antagonist G15 during beige adipocyte differentiation. G-1 and G15 treatment did not alter thermogenic gene expression in the GPRKO group (**Figure 4F**), however, antagonism of GPR30 significantly increased the gene expression of *Ucp-1* and *Prdm16* in WT-isolated SVF-differentiated beige adipocytes (**Figure 4G**). Collectively, these results demonstrated that GPR30 deficiency enhanced beige adipocyte differentiation by upregulating UCP-1, which may promote adipose tissue mitochondrial function and thereby protect against diet-induced adiposity.

### GPR30 Deficiency Promotes Adaptive Thermogenesis

**Figure 5:**
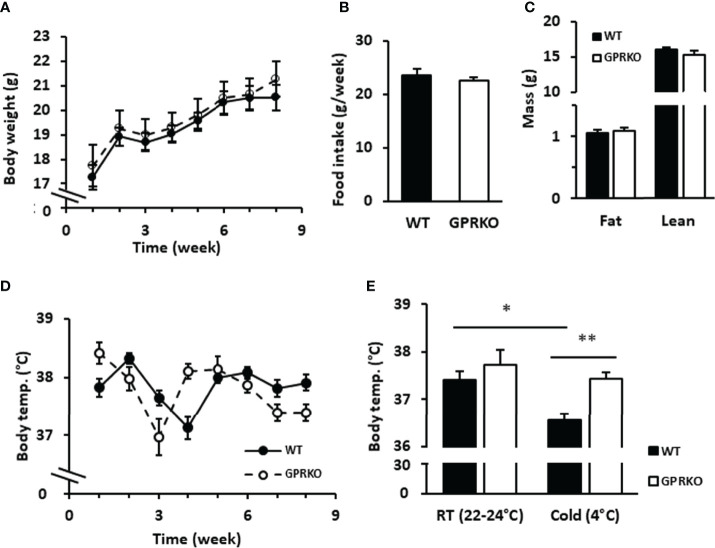
Deletion of GPR30 didn’t alter the regular rectal temperature but protected lean female mice from hypothermia. WT and GPRKO female mice were fed a standard chow diet (STD) for 8 weeks. **(A)** The body weight and **(B)** food intake were measured weekly. **(C)** The body composition were measured at week 7. **(D)** The weekly core body temperature was measured, and **(E)** the cold tolerance test were conducted at week 8 as described in Materials and Methods. Data are shown as means ± SE. n=7-10 mice/group. *p<0.05. **p<0.01.

Cold and overfeeding are the demonstrated stimuli activating nonshivering adaptive thermogenesis (10, 12, 49, 50). To further confirm whether the effect of GPR30 in thermogenic program *in vitro* is physiologically relevant, we performed a cold tolerance test using WT and GPRKO female mice. Consistent with our previous findings, the body weight, food intake, and body composition were similar between WT and GPRKO mice fed a STD diet (**Figures 5A**–**C**). The core body temperature of WT and GPRKO animals were constantly fluctuating without apparent pattern (**Figure 5D**). Mice were then exposed to the cold for 4 hours. As shown in **Figure 5E**, WT female mice had a reduced ability to defend their body temperature after 4-hour cold exposure, whereas deletion of GPR30 effectively protected female mice from developing hypothermia. Taken together, our results indicate for the first time, to our knowledge, that GPR30 is involved in regulating thermogenesis in response to either cold or overfeeding in female mice.

## Discussion

The present study provides evidence that GPR30 is a negative regulator of thermogenesis, which at least partially contributes to the reduced adiposity in the GPRKO female mice. First, UCP-1, the key regulator of brown and beige fat thermogenesis (8), is upregulated in the absence of GPR30. Second, the mitochondria in BAT are highly energetic in GPRKO animals while the WT mitochondria remain in a relatively quiescent stage. Third, deletion of GPR30 promotes the differentiation of beige adipocytes and induces BAT-specific gene transcription in WAT, and last, GPRKO increases adaptive thermogenesis in female mice exposed to cold. Together, our findings provide insight into the mechanism by which GPR30 regulates fat metabolism and adiposity in female mice exposed to excess calories.

It has been presumed that white and brown adipocytes are developmentally related (51), as they express common enzymes and, importantly, both require PPARγ for their differentiation (52, 53). While beige cells may have similar morphology and function as those of brown fat cells, it has been demonstrated that beige cells have a unique gene expression pattern different from that of either white or brown fat cells. Recently, the widely used adipocyte cell line, 3T3-L1 cells, have been reported to express the BAT-selective gene *Ucp-1* (54), suggesting that there is no common boundary between white and brown/beige adipocytes as previously thought. The 3T3-L1 cells can be induced to beige-like adipocytes with the treatment of rosiglitazone, T3, and IBMX, which are known inducers for beige differentiation (30). Like the white adipocytes, brown adipocytes and beige adipocytes store triglycerides in multilocular droplets, In addition to the differences in morphological structures and functions between WAT and BAT, there are distinct transcriptional factors involved in BAT development and thermogenesis (40, 55, 56), including *Sirt1*, *Pgc-1α*, *Prdm16*, etc. (46, 57, 58). *Pgc-1α* has been shown to induce transcription of numerous genes that comprise the thermogenic browning program, including *Ucp-1* and *Dio2* (59). However, we discovered that the mRNA levels of *Pgc-1α* were similar between WT and GPRKO mice (data not shown). It is notable that isolated brown adipocytes still express BAT-selective genes in the absence of *Pgc-1α* (60). Thus, the upregulation of UCP-1 in GPRKO adipose tissue may be *Pgc-1α*-independent.

It has been well known that thermogenic responses of brown and beige fat tissues in responses to cold exposure is primarily driven by the sympathetic nervous system (SNS) through the release of norepinephrine and subsequent activation of β-adrenergic receptor (βAR)-mediated signaling in fat cells (61). Therefore, βAR agonists are often used to mimic cold-induced SNS activity and subsequent thermogenesis program in cultured brown and beige adipocytes. However, we didn’t use βAR agonist to examine the effect of GPR30 deficiency on beige adipocyte differentiation, because activation of βAR may fully drive the beige cell thermogenesis in both WT and GPR30 KO cells, which could diminish the effect of GPR30 in the presence of the agonist, given that deletion of GPR30 increases cAMP production, beige cell differentiation, and thermogenesis, the same effects elicited by activating βAR. Thus, in present study, we evaluated GPR30-regulation of thermogenesis in beige cells under cold exposure, which was shown to directly stimulate thermogenic activity of white and beige cells in a βAR signaling-independent, cell-autonomous manner (32, 62).

We demonstrated that deletion of GPR30 greatly boosted the agonism of adenylyl cyclase, releasing cAMP in energy-enriched stromal cells. As reported before, mitochondrial cAMP signaling is an indispensable mechanism regulating mitochondrial energetics and homeostasis (63). Our result of CRE-luciferase activity assays clearly indicates that GPR30 deficiency indeed enhanced cAMP-regulated transcriptional activity.

In contrast to our previous finding that deletion of GPR30 only exerted metabolic effects in female but not male mice fed either a high fat diet or chow diet (24), a recent study reported that isoflavone genistein, a GPR30 agonist, increased adipose cAMP content, UCP-1 expression, and energy expenditure while reducing body weight and fat mass gain in wild-type male mice, but these effects were blunted in GPR30^-/-^ mice (64), suggesting that activation of GPR30 promotes thermogenic program in adipose tissue in male mice. These observations also contradict to our findings in the present study that inactivation of GPR30 increased cAMP production and thermogenic adipocyte differentiation in female mice. More studies are needed to investigate the reasons for causing these discrepancies, which could be due to the differences in gender (male vs. female mice), diet (high-fat sucrose vs. high-fat), and strain background (C57B6 vs. 129).

The primary function of mitochondria is to produce ATP as energy by using nutritional substrates (65). However, not all of the external energy supply is fully coupled to ATP synthesis. The extra energy is, thereby, dissipated as heat *via* the process of proton leak, which is regulated by the uncoupling proteins located on the inner membrane of mitochondria (66, 67). FCCP, a widely used mitochondrial uncoupler (68), stimulated oxygen consumption in WT adipose tissue, but it failed to manipulate the mitochondrial respiration of GPRKO BAT (data not shown). Given that GPR30 is a membrane-associated receptor and has been found in mitochondria in C2C12 cells (69) and breast cancer cells (70), it is reasonable to speculate that GPR30 localizes on the intermembrane of adipocyte mitochondria and blocks the activity of UCPs. Therefore, deletion of GPR30 elevates uncoupling protein activity and promotes thermogenesis, resulting in enhanced energy metabolism and reduced fat accumulation.

This study has some limitations. First, while we observed that deletion of GPR30 drastically increased *ucp-1* expression in beige cells exposed to cool temperature (31°C), it is unclear whether and to what extent this effect as observed *in vitro* contribute to the protective action of GPR30 inactivation against cold-induced hypothermia in mice, given that BAT may play a major role in thermogenesis through SNS-mediated activation of βAR (41). In that regard, the effects of GPR30 on beige and brown adipocyte thermogenesis under HFD feeding, the cold stress, and selective βAR agonism should be studied in the future research, which could be instrumental in defining cellular and molecular mechanisms by which inactivation of GPR30 promotes thermogenesis. Second, although WAT in GPRKO female mice displayed higher UCP-1 protein levels as compared with WT mice, we don’t have direct evidence that GPR30 deficiency increased the number of beige cells *in vivo*, which needs to be determined in future study. Third, while we were more focused on determining the effect of GPR30 on beige adipogenesis, this study should have included examining the effect of GPR30 on cAMP signaling and brown adipocytes of BAT-derived SVF cells, given the critical role of BAT in thermogenesis. Lastly, in the present study, whole body GPRKO mice were used for investigating the metabolic role of GPR30 in adipose tissues. While it is preferable to use conditional GPRKO mice for determining its tissue-specific effects, genetic tools to specifically target adipocyte precursor cells for studying adipogenesis are unavailable, as the use of presently available fat-specific Cre mouse lines (such as adiponectin-driven Cre recombinase mice) can only delete GPR30 in mature adipocytes (71).

## Conclusion

In summary, we previously revealed the effects of GPR30 on energy metabolism and fat mass control in female mice exposed to a HFD. Here, we further demonstrate that deletion of *GPR30* protects female mice from hypothermia and promotes the mitochondrial energetic phenotype in female BAT as compared to WT littermates. Consequently, deletion of GPR30 enhances subcutaneous WAT-isolated SVF differentiating to beige adipocytes and activates the thermogenic beiging program through up-regulation of UCP-1, which thereby protects female mice from HFD-induced obesity. Further work will be required to delineate the detailed mechanism of GPR30-regulated beige adipogenesis, body weight control, as well as the possible interaction between GPR30 and ERα-mediated metabolic actions, which could potentially lead to a novel therapeutic strategy to efficiently prevent the development of obesity and obesity related metabolic diseases in females.

## Data Availability Statement

The raw data supporting the conclusions of this article will be made available by the authors, without undue reservation.

## Ethics Statement

The animal study was reviewed and approved by Institutional Animal Care and Use Committee at Virginia Tech.

## Author Contributions

JL, conceptualization, methodology, investigation, validation, formal analysis, writing - original draft, writing - review and editing. YW, investigation and validation. EG, resources, writing - review and editing. DL, conceptualization, methodology, writing - review and editing, supervision, project administration, and funding acquisition. All authors contributed to the article and approved the submitted version.

## Funding

This work was partially supported by a seed grant from Virginia Tech, a grant from Diabetes Action Research and Education Foundation (USA), a grant from Virginia Tech Drug Discovery Center, and a Virginia Tech Pratt Fellowship Award.

## Conflict of Interest

The authors declare that the research was conducted in the absence of any commercial or financial relationships that could be construed as a potential conflict of interest.

## Publisher’s Note

All claims expressed in this article are solely those of the authors and do not necessarily represent those of their affiliated organizations, or those of the publisher, the editors and the reviewers. Any product that may be evaluated in this article, or claim that may be made by its manufacturer, is not guaranteed or endorsed by the publisher.

## Abbreviations

Ab/Am, antibiotic antimycotic; AC, adenylate cyclase; ASM, acid soluble metabolites; BAT, brown adipose tissue; BSA, bovine serum albumin; BW, body weight; cAMP, cyclic adenosine monophosphate; CRE, cAMP response element; ECAR, extracellular acidification rate; FBS, fetal bovine serum; GPCRs, G protein-coupled receptors; GPR30, G protein-coupled receptor 30; GPRKO, GPR30 knockout; HFD, high-fat diet; IBMX, 3-isobutyl-1-methylxanthine; KRB, Krebs-Ringer bicarbonate buffer; OCR, oxygen consumption rate; PET, positron emission tomography; PKA, protein kinase A; PPARγ, peroxisome proliferator activated receptor γ; RT, room temperature; STD, standard chow diet; SVF, stromal vascular fraction; T3, triiodothyronine; UCP-1, uncoupling protein 1; WAT, white adipose tissue.

## References

[1] N.C.D.R.F. Collaboration. Worldwide Trends in Body-Mass Index, Underweight, Overweight, and Obesity From 1975 to 2016: A Pooled Analysis of 2416 Population-Based Measurement Studies in 128.9 Million Children, Adolescents, and Adults. Lancet (2017) 390:2627–42. doi: 10.1016/S0140-6736(17)32129-3 PMC5735219 29029897

[2] Twig G Yaniv G Levine H Leiba A Goldberger N Derazne E. Body-Mass Index in 2.3 Million Adolescents and Cardiovascular Death in Adulthood. N Engl J Med (2016) 374:2430–40. doi: 10.1056/NEJMoa1503840 27074389

[3] Svensson KJ Long JZ Jedrychowski MP Cohen P Lo JC Serag S. A Secreted Slit2 Fragment Regulates Adipose Tissue Thermogenesis and Metabolic Function. Cell Metab (2016) 23:454–66. doi: 10.1016/j.cmet.2016.01.008 PMC4785066 26876562

[4] Blüher M. Obesity: Global Epidemiology and Pathogenesis. Nat Rev Endocrinol (2019) 15:288–98. doi: 10.1038/s41574-019-0176-8 30814686

[5] Romieu I Dossus L Barquera S Blottière HM Franks PW Gunter M. Energy Balance and Obesity: What are the Main Drivers? Cancer Causes Control (2017) 28:247–58. doi: 10.1007/s10552-017-0869-z PMC5325830 28210884

[6] Bray G Kim K Wilding J Federation WO. Obesity: A Chronic Relapsing Progressive Disease Process. A Position Statement of the World Obesity Federation. Obes Rev (2017) 18:715–23. doi: 10.1111/obr.12551 28489290

[7] Bray GA. From Farm to Fat Cell: Why Aren’t We All Fat? Metab Clin Exp (2015) 64:349–53. doi: 10.1016/j.metabol.2014.09.012 25554523

[8] Kissig M Shapira SN Seale P. SnapShot: Brown and Beige Adipose Thermogenesis. Cell (2016) 166:258–258.e1. doi: 10.1016/j.cell.2016.06.038 27368105 PMC5478388

[9] Virtanen KA Lidell ME Orava J Heglind M Westergren R Niemi T. Functional Brown Adipose Tissue in Healthy Adults. N Engl J Med (2009) 360:1518–25. doi: 10.1056/NEJMoa0808949 19357407

[10] Wu J Bostrom P Sparks LM Ye L Choi JH Giang AH. Beige Adipocytes are a Distinct Type of Thermogenic Fat Cell in Mouse and Human. Cell (2012) 150:366–76. doi: 10.1016/j.cell.2012.05.016 PMC3402601 22796012

[11] Kim SH Plutzky J. Brown Fat and Browning for the Treatment of Obesity and Related Metabolic Disorders. Diabetes Metab J (2016) 40:12–21. doi: 10.4093/dmj.2016.40.1.12 26912151 PMC4768046

[12] Rothwell NJ Stock MJ. A Role for Brown Adipose Tissue in Diet-Induced Thermogenesis. Nature (1979) 281:31–5. doi: 10.1038/281031a0 551265

[13] Rosen ED Spiegelman BM. What We Talk About When We Talk About Fat. Cell (2014) 156:20–44. doi: 10.1016/j.cell.2013.12.012 24439368 PMC3934003

[14] Chondronikola M Volpi E Børsheim E Porter C Saraf MK Annamalai P. Brown Adipose Tissue Activation Is Linked to Distinct Systemic Effects on Lipid Metabolism in Humans. Cell Metab (2016) 23:1200–6. doi: 10.1016/j.cmet.2016.04.029 PMC4967557 27238638

[15] Caron A Reynolds RP Castorena CM Michael NJ Lee CE Lee S. Adipocyte Gs But Not Gi Signaling Regulates Whole-Body Glucose Homeostasis. Mol Metab (2019) 27:11–21. doi: 10.1016/j.molmet.2019.06.019 31279640 PMC6717754

[16] Collins S. β-Adrenoceptor Signaling Networks in Adipocytes for Recruiting Stored Fat and Energy Expenditure. Front Endocrinol (2011) 2:102. doi: 10.3389/fendo.2011.00102 PMC3355892 22654837

[17] Klepac K Kilić A Gnad T Brown LM Herrmann B Wilderman A. The Gq Signalling Pathway Inhibits Brown and Beige Adipose Tissue. Nat Commun (2016) 7:10895. doi: 10.1038/ncomms10895 26955961 PMC4786868

[18] Kobilka BK. G Protein Coupled Receptor Structure and Activation. Biochim Biophys Acta (BBA) - Biomembr (2007) 1768:794–807. doi: 10.1016/j.bbamem.2006.10.021 PMC1876727 17188232

[19] Wettschureck N Offermanns S. Mammalian G Proteins and Their Cell Type Specific Functions. Physiol Rev (2005) 85:1159–204. doi: 10.1152/physrev.00003.2005 16183910

[20] Kazak L Chouchani ET Jedrychowski MP Erickson BK Shinoda K Cohen P. A Creatine-Driven Substrate Cycle Enhances Energy Expenditure and Thermogenesis in Beige Fat. Cell (2015) 163:643–55. doi: 10.1016/j.cell.2015.09.035 PMC4656041 26496606

[21] Mills EL Pierce KA Jedrychowski MP Garrity R Winther S Vidoni S. Accumulation of Succinate Controls Activation of Adipose Tissue Thermogenesis. Nature (2018) 560:102–+. doi: 10.1038/s41586-018-0353-2 PMC7045287 30022159

[22] Shabalina IG Petrovic N de Jong JMA Kalinovich AV Cannon B Nedergaard J. UCP1 in Brite/Beige Adipose Tissue Mitochondria Is Functionally Thermogenic. Cell Rep (2013) 5:1196–203. doi: 10.1016/j.celrep.2013.10.044 24290753

[23] Luo J Liu DM. Does GPER Really Function as a G Protein-Coupled Estrogen Receptor *In Vivo* ? Front Endocrinol (2020) 11. doi: 10.3389/fendo.2020.00148 PMC7137379 32296387

[24] Wang A Luo J Moore W Alkhalidy H Wu L Zhang J. GPR30 Regulates Diet-Induced Adiposity in Female Mice and Adipogenesis *In Vitro* . Sci Rep (2016) 6:34302. doi: 10.1038/srep34302 27698362 PMC5048424

[25] Dunham-Snary KJ Sandel MW Westbrook DG Ballinger SW. A Method for Assessing Mitochondrial Bioenergetics in Whole White Adipose Tissues. Redox Biol (2014) 2:656–60. doi: 10.1016/j.redox.2014.04.005 PMC4052527 24936439

[26] Goswami I Perry JB Allen ME Brown DA von Spakovsky MR Verbridge SS. Influence of Pulsed Electric Fields and Mitochondria-Cytoskeleton Interactions on Cell Respiration. Biophys J (2018) 114:2951–64. doi: 10.1016/j.bpj.2018.04.047 PMC6026445 29925031

[27] Yu G Wu X Kilroy G Halvorsen YD Gimble JM Floyd ZE. Isolation of Murine Adipose-Derived Stem Cells. Methods Mol Biol (2011) 702:29–36. doi: 10.1007/978-1-61737-960-4_3 21082392

[28] Liu D Zhen W Yang Z Carter JD Si H Reynolds KA. Genistein Acutely Stimulates Insulin Secretion in Pancreatic Beta-Cells Through a cAMP-Dependent Protein Kinase Pathway. Diabetes (2006) 55:1043–50. doi: 10.2337/diabetes.55.04.06.db05-1089 16567527

[29] Luo J Wang A Zhen W Wang Y Si H Jia Z. Phytonutrient Genistein is a Survival Factor for Pancreatic Beta-Cells *via* GPR30-Mediated Mechanism. J Nutr Biochem (2018) 58:59–70. doi: 10.1016/j.jnutbio.2018.04.018 29885598 PMC6095734

[30] Aune UL Ruiz L Kajimura S. Isolation and Differentiation of Stromal Vascular Cells to Beige/Brite Cells. J Vis Exp (2013) 28(73):50191. doi: 10.3791/50191 PMC3641667 23568137

[31] Seale P Kajimura S Yang W Chin S Rohas LM Uldry M. Transcriptional Control of Brown Fat Determination by PRDM16. Cell Metab (2007) 6:38–54. doi: 10.1016/j.cmet.2007.06.001 17618855 PMC2564846

[32] Lugo Leija HA Velickovic K Bloor I Sacks H Symonds ME Sottile V. Cold-Induced Beigeing of Stem Cell-Derived Adipocytes Is Not Fully Reversible After Return to Normothermia. J Cell Mol Med (2020) 24:11434–44. doi: 10.1111/jcmm.15749 PMC7576274 32902117

[33] Liu DM Iruthayanathan M Homan LL Wang YQ Yang LL Wang Y. Dehydroepiandrosterone Stimulates Endothelial Proliferation and Angiogenesis Through Extracellular Signal-Regulated Kinase 1/2-Mediated Mechanisms. Endocrinology (2008) 149:889–98. doi: 10.1210/en.2007-1125 PMC2275364 18079198

[34] Li X Luo J Anandh Babu PV Zhang W Gilbert E Cline M. Dietary Supplementation of Chinese Ginseng Prevents Obesity and Metabolic Syndrome in High-Fat Diet-Fed Mice. J Med Food (2014) 17:1287–97. doi: 10.1089/jmf.2014.0016 PMC4259183 25076190

[35] Cheng G Zielonka J McAllister D Tsai S Dwinell MB Kalyanaraman B. Profiling and Targeting of Cellular Bioenergetics: Inhibition of Pancreatic Cancer Cell Proliferation. Br J Cancer (2014) 111:85–93. doi: 10.1038/bjc.2014.272 24867695 PMC4090735

[36] Diers AR Vayalil PK Oliva CR Griguer CE Darley-Usmar V Hurst DR. Mitochondrial Bioenergetics of Metastatic Breast Cancer Cells in Response to Dynamic Changes in Oxygen Tension: Effects of HIF-1α. PloS One (2013) 8:e68348. doi: 10.1371/journal.pone.0068348 23840849 PMC3696014

[37] Dott W Mistry P Wright J Cain K Herbert KE. Modulation of Mitochondrial Bioenergetics in a Skeletal Muscle Cell Line Model of Mitochondrial Toxicity. Redox Biol (2014) 2:224–33. doi: 10.1016/j.redox.2013.12.028 PMC3909783 24494197

[38] Perry JB Davis GN Allen ME Makrecka-Kuka M Dambrova M Grange RW. Cardioprotective Effects of Idebenone do Not Involve ROS Scavenging: Evidence for Mitochondrial Complex I Bypass in Ischemia/Reperfusion Injury. J Mol Cell Cardiol (2019) 135:160–71. doi: 10.1016/j.yjmcc.2019.08.010 31445917

[39] Jastroch M Hirschberg V Klingenspor M. Functional Characterization of UCP1 in Mammalian HEK293 Cells Excludes Mitochondrial Uncoupling Artefacts and Reveals No Contribution to Basal Proton Leak. Bba-Bioenergetics (2012) 1817:1660–70. doi: 10.1016/j.bbabio.2012.05.014 22676960

[40] Harms M Seale P. Brown and Beige Fat: Development, Function and Therapeutic Potential. Nat Med (2013) 19:1252–63. doi: 10.1038/nm.3361 24100998

[41] Cannon B Nedergaard J. Brown Adipose Tissue: Function and Physiological Significance. Physiol Rev (2004) 84:277–359. doi: 10.1152/physrev.00015.2003 14715917

[42] Nedergaard J Cannon B. The Browning of White Adipose Tissue: Some Burning Issues. Cell Metab (2014) 20:396–407. doi: 10.1016/j.cmet.2014.07.005 25127354

[43] Bartelt A Heeren J. Adipose Tissue Browning and Metabolic Health. Nat Rev Endocrinol (2014) 10:24–36. doi: 10.1038/nrendo.2013.204 24146030

[44] Seale P Conroe HM Estall J Kajimura S Frontini A Ishibashi J. Prdm16 Determines the Thermogenic Program of Subcutaneous White Adipose Tissue in Mice. J Clin Invest (2011) 121:96–105. doi: 10.1172/JCI44271 21123942 PMC3007155

[45] Lee YH Jung YS Choi D. Recent Advance in Brown Adipose Physiology and its Therapeutic Potential. Exp Mol Med (2014) 46:e78. doi: 10.1038/emm.2013.163 24556827 PMC3944445

[46] Ohno H Shinoda K Spiegelman BM Kajimura S. PPAR Gamma Agonists Induce a White-To-Brown Fat Conversion Through Stabilization of PRDM16 Protein. Cell Metab (2012) 15:395–404. doi: 10.1016/j.cmet.2012.01.019 22405074 PMC3410936

[47] Roberts LD Ashmore T Kotwica AO Murfitt SA Fernandez BO Feelisch M. Inorganic Nitrate Promotes the Browning of White Adipose Tissue Through the Nitrate-Nitrite-Nitric Oxide Pathway. Diabetes (2015) 64:471–84. doi: 10.2337/db14-0496 PMC4351918 25249574

[48] Qiang L Wang L Kon N Zhao W Lee S Zhang Y. Brown Remodeling of White Adipose Tissue by SirT1-Dependent Deacetylation of Pparγ. Cell (2012) 150:620–32. doi: 10.1016/j.cell.2012.06.027 PMC3413172 22863012

[49] Cousin B Cinti S Morroni M Raimbault S Ricquier D Penicaud L. Occurrence of Brown Adipocytes in Rat White Adipose Tissue: Molecular and Morphological Characterization. J Cell Sci (1992) 103(Pt 4):931–42. doi: 10.1242/jcs.103.4.931 1362571

[50] Hamann A Flier JS Lowell BB. Decreased Brown Fat Markedly Enhances Susceptibility to Diet-Induced Obesity, Diabetes, and Hyperlipidemia. Endocrinology (1996) 137:21–9. doi: 10.1210/endo.137.1.8536614 8536614

[51] Gesta S Tseng YH Kahn CR. Developmental Origin of Fat: Tracking Obesity to Its Source. Cell (2007) 131:242–56. doi: 10.1016/j.cell.2007.10.004 17956727

[52] Hansen JB Kristiansen K. Regulatory Circuits Controlling White Versus Brown Adipocyte Differentiation. Biochem J (2006) 398:153–68. doi: 10.1042/BJ20060402 PMC1550312 16898874

[53] Rosen ED Spiegelman BM. Molecular Regulation of Adipogenesis. Annu Rev Cell Dev Biol (2000) 16:145–71. doi: 10.1146/annurev.cellbio.16.1.145 11031233

[54] Asano H Kanamori Y Higurashi S Nara T Kato K Matsui T. Induction of Beige-Like Adipocytes in 3T3-L1 Cells. J Vet Med Sci (2014) 76:57–64. doi: 10.1292/jvms.13-0359 24065084 PMC3979956

[55] Villarroya F Vidal-Puig A. Beyond the Sympathetic Tone: The New Brown Fat Activators. Cell Metab (2013) 17:638–43. doi: 10.1016/j.cmet.2013.02.020 23583169

[56] Lee YH Mottillo EP Granneman JG. Adipose Tissue Plasticity From WAT to BAT and in Between. Bba-Mol Basis Dis (2014) 1842:358–69. doi: 10.1016/j.bbadis.2013.05.011 PMC4435780 23688783

[57] Qiang L Wang L Kon N Zhao W Lee S Zhang Y. Brown Remodeling of White Adipose Tissue by SirT1-Dependent Deacetylation of Ppargamma. Cell (2012) 150:620–32. doi: 10.1016/j.cell.2012.06.027 PMC3413172 22863012

[58] Kajimura S. Promoting Brown and Beige Adipocyte Biogenesis Through the PRDM16 Pathway. Int J Obes Suppl (2015) 5:S11. doi: 10.1038/ijosup.2015.4 27152168 PMC4850573

[59] Puigserver P Adelmant G Wu Z Fan M Xu J O'malley B. Activation of Pparγ Coactivator-1 Through Transcription Factor Docking. Science (1999) 286:1368–71. doi: 10.1126/science.286.5443.1368 10558993

[60] Uldry M Yang W St-Pierre J Lin J Seale P Spiegelman BM. Complementary Action of the PGC-1 Coactivators in Mitochondrial Biogenesis and Brown Fat Differentiation. Cell Metab (2006) 3:333–41. doi: 10.1016/j.cmet.2006.04.002 16679291

[61] Ueta CB Fernandes GW Capelo LP Fonseca TL Maculan FD Gouveia CH. Beta(1) Adrenergic Receptor is Key to Cold- and Diet-Induced Thermogenesis in Mice. J Endocrinol (2012) 214:359–65. doi: 10.1530/JOE-12-0155 PMC4977996 22728333

[62] Ye L Wu J Cohen P Kazak L Khandekar MJ Jedrychowski MP. Fat Cells Directly Sense Temperature to Activate Thermogenesis. Proc Natl Acad Sci USA (2013) 110:12480–5. doi: 10.1073/pnas.1310261110 PMC3725077 23818608

[63] Ould Amer Y Hebert-Chatelain E. Mitochondrial cAMP-PKA Signaling: What do We Really Know? Biochim Biophys Acta Bioenerg (2018) 1859:868–77. doi: 10.1016/j.bbabio.2018.04.005 29694829

[64] Vásquez-Reyes S Vargas-Castillo A Noriega LG Velázquez-Villegas LA Pérez B Sánchez-Tapia M. Genistein Stimulation of White Adipose Tissue Thermogenesis Is Partially Dependent on GPR30 in Mice. Mol Nutr Food Res (2022) 66(8):e2100838. doi: 10.1002/mnfr.202100838 35142428 PMC10205097

[65] Mitchell P. Coupling of Phosphorylation to Electron and Hydrogen Transfer by a Chemi-Osmotic Type of Mechanism. Nature (1961) 191:144–8. doi: 10.1038/191144a0 13771349

[66] Brand MD Affourtit C Esteves TC Green K Lambert AJ Miwa S. Mitochondrial Superoxide: Production, Biological Effects, and Activation of Uncoupling Proteins. Free Radic Biol Med (2004) 37:755–67. doi: 10.1016/j.freeradbiomed.2004.05.034 15304252

[67] Krauss S Zhang CY Lowell BB. The Mitochondrial Uncoupling-Protein Homologues. Nat Rev Mol Cell Biol (2005) 6:248–61. doi: 10.1038/nrm1592 15738989

[68] Heytler PG. Uncouplers of Oxidative Phosphorylation. Methods Enzymol (1979) 55:462–42. doi: 10.1016/0076-6879(79)55060-5 156853

[69] Ronda AC Boland RL. Intracellular Distribution and Involvement of GPR30 in the Actions of E2 on C2C12 Cells. J Cell Biochem (2016) 117:793–805. doi: 10.1002/jcb.25369 26359786

[70] Wei W Chen ZJ Zhang KS Yang XL Wu YM Chen XH. The Activation of G Protein-Coupled Receptor 30 (GPR30) Inhibits Proliferation of Estrogen Receptor-Negative Breast Cancer Cells *In Vitro* and *In Vivo* . Cell Death Dis (2014) 5:e1428. doi: 10.1038/cddis.2014.398 25275589 PMC4649509

[71] Jeffery E Berry R Church CD Yu S Shook BA Horsley V. Characterization of Cre Recombinase Models for the Study of Adipose Tissue. Adipocyte (2014) 3:206–11. doi: 10.4161/adip.29674 PMC4110097 25068087

